# The development of an immersive mixed-reality application to improve the ecological validity of eating and sensory behavior research

**DOI:** 10.3389/fnut.2023.1170311

**Published:** 2023-07-19

**Authors:** John W. Long, Bart Masters, Pejman Sajjadi, Christopher Simons, Travis D. Masterson

**Affiliations:** ^1^Department of Nutritional Sciences, The Pennsylvania State University, University Park, PA, United States; ^2^The Center for Immersive Experiences, The Pennsylvania State University, University Park, PA, United States; ^3^Department of Software Engineering and Game Development, Kennesaw State University, Marietta, GA, United States; ^4^Department of Food Science and Technology, The Ohio State University, Columbus, OH, United States

**Keywords:** virtual reality, extended reality, augmented reality, mixed reality, immersive technology, ingestive behavior, sensory evaluation

## Abstract

**Introduction:**

The modern eating environment has been implicated as a driving force of the obesity epidemic. Mixed reality applications may improve traditional methodological assessments of eating behavior by improving the ecological validity of the laboratory setting.

**Methods:**

Research experts evaluated the utility and ecological validity of a mixed reality application that allowed immersion within virtual environments through utilizing the passthrough cameras of the head mounted display to view and interact with real foods. An initial evaluation was conducted that involved three virtual environments: a traditional laboratory booth, a non-textured restaurant, and a full-textured restaurant. The feedback from the initial evaluation was used to create a new virtual restaurant environment and a subsequent evaluation was conducted.

**Results:**

Nearly all research experts suggested adding social cues such as people and background noise to create a more authentic and ecologically valid experience. The experts scored the new virtual restaurant environment to be more acceptable than eating or conducting research in a sensory booth but scored lower when compared to conducting research in a real-world restaurant setting.

**Discussion:**

The results of this evaluation suggest that mixed reality applications may be a new methodology to assess environmental influences of eating behavior and may be a promising direction for eating behavior and sensory science research.

## Introduction

The increasing prevalence of obesity worldwide highlights the need to better understand food choices and eating behaviors (1). Currently, laboratory-assessed eating behavior is the principal method of researching eating behavior and its connection to health. Laboratory environments offer a great degree of internal validity by providing control over a variety of important variables related to eating behaviors (2). However, the laboratory eating environment (e.g., an isolated, sterile sensory booth) is not representative of the eating environment in the real world (2). This is likely to limit the external validity of the study’s findings as the observed eating behaviors may not be completely reflective of eating behaviors under “real-world” conditions (3).

Human eating behaviors are strongly influenced by both internal personal factors and by external environmental factors (4, 5). A variety of external environmental factors have been shown to increase food consumption including distraction-related factors (6) (e.g., screens) social factors (7) (e.g., eating in the presence of others), and visual cues (8, 9) (e.g., food advertising). Additionally, the hedonic response to food can differ depending on the setting or location (3, 10). These external environmental factors provide a stimulus that evokes an important internal factor known as food cue reactivity (5). Food cue reactivity refers to the psychological and physiological responses evoked by environmental cues, such as food advertising, and encompasses the heightened attention, salivation, craving, and desire to consume food (5). These strong external factors are typically absent from the typical sterile laboratory environment. This can increase experimental control but also reduces ecological validity. Attempting to conduct research in real-world settings is also challenging as gaining access to, manipulating, and controlling relevant variables of interest is difficult. Researchers have gone to great lengths to attempt to replicate real-world eating situations to improve the ecological validity of the laboratory environment. For example, some researchers have created simulated fast food restaurants or large-scale canteens (11, 12). This has allowed these labs to investigate eating behavior in a more ecologically valid manner but at a great cost in terms of money, time, scalability, and resources (13, 14).

One solution to these issues may lie in the use of Extended Reality technologies. Extended Reality is an umbrella term referring to the different technologies that either extend our reality (i.e., Augmented/Mixed Reality) or replace reality with an entirely virtual experience (i.e., Virtual Reality). Mixed Reality (MR) in particular could serve as a great tool for improving the ecological validity of research settings due to its unique characteristics. While there is no consensus as what MR is, there are operational definitions for this term in the literature (15). For instance, Pan et al., have defined MR as “*the incorporation of virtual computer graphics objects into a real three dimensional scene, or alternatively the inclusion of real world elements into a virtual environment*” ((16), page 1). The first part of this operational definition refers mostly to augmented reality, whereas the second part refers to what is known as augmented virtuality. This definition is in line with what is labeled as “Strong Augmented Reality” by many researchers in the community [for an overview, refer to Speicher et al. (15)]. On similar grounds, MR is also defined as a hybrid-experience in which a user can simultaneously see and interact with real world objects while they are immersed within a virtual environment (15).

In theory, MR can improve the ecological validity of the laboratory by modifying the immediate physical environment to be more representative of authentic real-world environments (17). Mixed Reality in this sense can potentially provide the best of both worlds. On the one hand, MR can afford researchers to take advantage of the rigor and control in traditional testing environments by incorporating the essential components of the research as tangible real-world objects. On the other hand, MR can afford researchers the ability to modify environmental cues that are often difficult/impossible to be manipulated in traditional testing environments by presenting them as virtual objects (18).

One of the key characteristics of extended reality technologies is immersion. The term immersion is an objective measure that refers to the extent to which a technology can present a vivid virtual environment while separating the user from their immediate physical reality (19). An effective immersive experience induces a sense of presence or “being there” by allowing a user to experience a computer-generated world as if it were genuine (20). The term presence refers to an individual’s subjective perception of immersion and is one of the key features researchers aim to provide the user in order to fill the gap between laboratory and natural contexts (19, 20).

Extended Reality technologies vary significantly in their level of immersion depending on their display and interactivity characteristics (21). Currently, Head-Mounted Displays provide the highest level of immersion as the experience is completely wrapped around the user (i.e., 360°), allowing the user to experience and interact with the displayed virtual environment in a nearly 1:1 aspect ratio while simultaneously completely blocking out the real-world environment (22, 23). This illusion is created effortlessly in immersive Virtual Reality (iVR) experiences. However, with MR, due to a mix of virtual and physical objects, immersion can break. This is particularly the case with MR headsets such as the HoloLens^1^ as a result of the very limited field of view of the device (54°), making the experience very difficult to elicit the feeling of “being there.” Small field of view (compared to modern iVR headsets with an average field of view of 90°−120°) makes it rather difficult to integrate real world elements into a virtual environment in a meaningful way, as was suggested by Pan and colleagues (16). We have witnessed, however, significant advancements in the extended reality community with the introduction of Meta Quest 2 and later the Meta Quest Pro^2^ in 2022. While these head mounted displays were originally intended for VR experiences, they are also equipped with passthrough cameras that can turn the headset into a MR device. The significantly larger field of view of iVR head mounted displays can be potentially used for creating immersive MR applications where the experience is predominantly virtual with elements of the real world. According to Speicher et al., however, the relationship between the level of virtuality and immersion is not linear (15). In other words, one can populate an MR experience with virtual objects, but if the virtual objects do not meaningfully interact with the real-world objects, users may not feel immersed. As such, interactivity as a mechanism that facilitates immersion becomes very important in MR.

Other technologies such as video wall systems would be considered semi-immersive as they do not completely block out the real-world surrounding and therefore hardware such as video projectors may be evident (24). Additionally, depth perception of the projected images is limited and objects on the screens cannot be interacted with in a realistic manner. The lowest levels of immersive technologies typically utilize a computer screen or phone screen and are limited to the inability to block out the surrounding environment, display size, field-of-view, and the resolution of the screen (22). Therefore, highly immersive technologies are more effective at creating a laboratory environment that is representative of a natural context.

Immersive and semi-immersive technologies have been utilized in a variety of research domains such as food choice (25, 26), product perception (27, 28), sensory evaluation (29–31), nutrition education (32, 33), food cravings and food cues (34–36), food shopping behavior (37, 38), and eating disorders (39–41). For example, video wall systems have been used to improve consumer testing through improving the ecological validity and engagement of the laboratory environment by depicting a virtual coffeehouse (42). This study found differences in the liking and preference order for coffees evaluated in a virtual coffeehouse versus a traditional sensory booth, as well as the virtual coffeehouse to be a more reliable predictor of future coffee liking (42). Similarly, immersing individuals to a virtual video wall farm patio overlooking the countryside has been shown to impact liking ratings of vegetable products (31). These results suggest that the traditional laboratory environment lacks the extrinsic contextual information that is used in conjunction with the intrinsic attributes of the food to shape the hedonic response of the food.

The current environments used in sensory and consumer research have been criticized for the lack of ecological validity, while virtual reality and immersive technologies have been highlighted for their potential to improve the ecological validity of testing environments (17, 43). The implementation of VR in consumer research has been used particularly to explore food choice and food purchasing behavior in virtual environments such as virtual supermarkets. Behavior in virtual supermarkets has been shown to be highly related and correlated to real-life behavior (22). Additionally, these virtual supermarkets have been used to validate and investigate the potential of cues and nudges to prompt consumers to choose healthier products (18, 44, 45). The strong correlation between behavior in virtual and real-life settings highlights the utility of VR as a tool for researching food choice and purchasing behavior within ecologically valid testing environments.

Immersive virtual reality provides the opportunity to overcome some of the aforementioned challenges and limitations in relation to the ecological validity of research studies. Immersive VR allows a user to experience a computer-generated environment from a first-person perspective through a head mounted display (i.e., embodiment (46)) and continuously updates the experience in real time to allow for a congruency between the computer-generated environment and the user’s head and body movements (19, 47). Virtual reality has been used to assess whether exposure to cues of high-calorie and highly palatable food has an effect on reported food craving by immersing participants in virtual environments with low-calorie and high-calorie food cues (34). The study found that virtual environments with high-calorie food cues elicited higher levels of craving, indicating that food cue exposure in virtual environments induced food craving (34). For example, VR has been previously used to assess the effect of the eating environment on food intake and eating behavior by immersing participants in a virtual pizza restaurant (48). Participants reported a greater sense of presence in the virtual pizza restaurant compared to the laboratory setting. Similarly, another project found that an immersive virtual café elicited the most similar level of presence to a real café when compared to other methodologies (49).

While iVR has been shown to improve a sense of presence and therefore ecological validity, its usage within eating behavior research has been limited due to the inability to see or interact with actual food stimuli while immersed. With MR, however, participants can simultaneously see and interact with real world objects (i.e., actual food) while they are immersed within a virtual environment. This allows for the participants to be placed within a more ecologically relevant virtual environment, that can be tightly controlled and manipulated, while allowing participants to see, interact, and consume actual food. The aim of the present study is to detail the development and design of a mixed-reality application to allow users to be placed in a fully immersive environment within a laboratory setting while allowing them to see, interact with, and consume food. The virtual environments were evaluated by research experts to determine the potential utility, ecological validity, and relevance to eating behavior and sensory-related research. This evaluation aimed to assess whether the virtual environments warranted further development and to determine the utility of the technology in eating behavior and sensory-related research. Due to the exploratory nature of the study and the focus on technological innovation, no specific hypotheses were formulated.

## Materials and methods

### Overview

An initial application was designed that utilized the Meta Quest 2 head mounted display. The passthrough cameras used by this specific headset presented the user with black and white visuals of real-world objects within the virtual environment. However, this initial prototype application was designed in preparation for technological advances that would allow for full color passthrough while also allowing us to begin designing a virtual environment for future studies. The initial application was evaluated and received feedback based on the potential utility, ecological validity, and relevance to eating behavior and sensory-related research (see Study 1 below). An updated application and virtual restaurant were subsequently designed and a follow-up evaluation was conducted (see Study 2 below). This second iteration of testing was conducted using the Meta Quest Pro which utilized full color pass through cameras allowing full functionality of the application. This project was classified as program evaluation and quality improvement and therefore did not meet the definition of human subjects’ research as such it did not qualify for full Institutional Review Board Review.

### Integration of passthrough cameras with the virtual environments

Passthrough virtual reality is a feature that utilizes the head mounted display’s external cameras to capture the real-world environment around the user and display the real word inside the headset. To integrate real-world objects perceived through the passthrough camera into the virtual experience, we devised passthrough projection surfaces using a specific geometry. This meant selecting certain surfaces, planes, and 3D objects to display the external camera feed on their surfaces in place of the usual textures and materials that would normally be displayed in a fully virtual environment. The preliminary implementation of passthrough VR in this project used two flat passthrough planes attached to a virtual tabletop in the virtual environment. The planes were placed at a right angle to each other, positioned with one flat against the virtual tabletop and the other standing perpendicular to the virtual tabletop. This allowed the user to see the real-world tabletop space through the passthrough plane and any object placed on it as if it they were part of the virtual tabletop.

**Figure 1:**
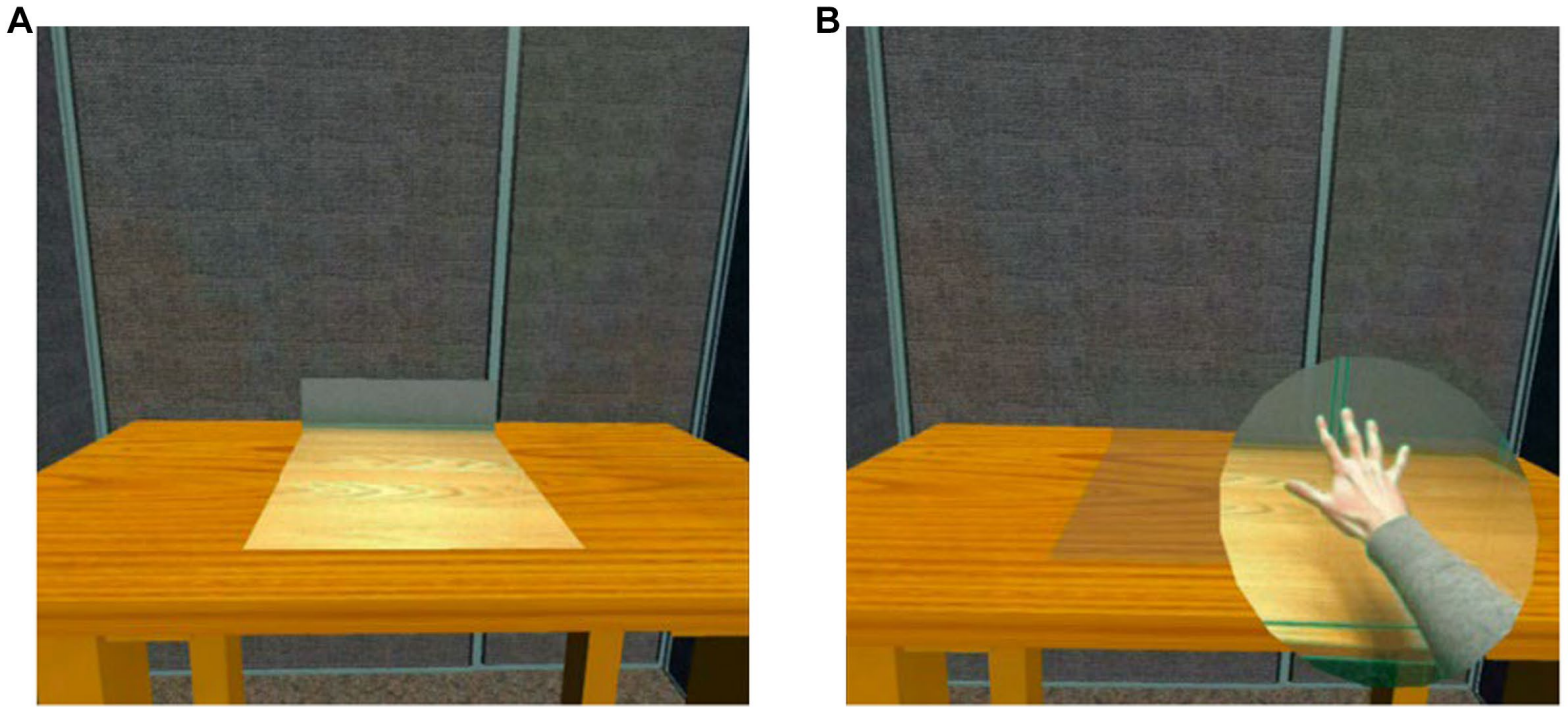
**(A)** Tabletop passthrough plane enabled when hand discs passthrough is absent, **(B)** Tabletop passthrough plane disabled when hand discs passthrough is present.

**Figure 2:**
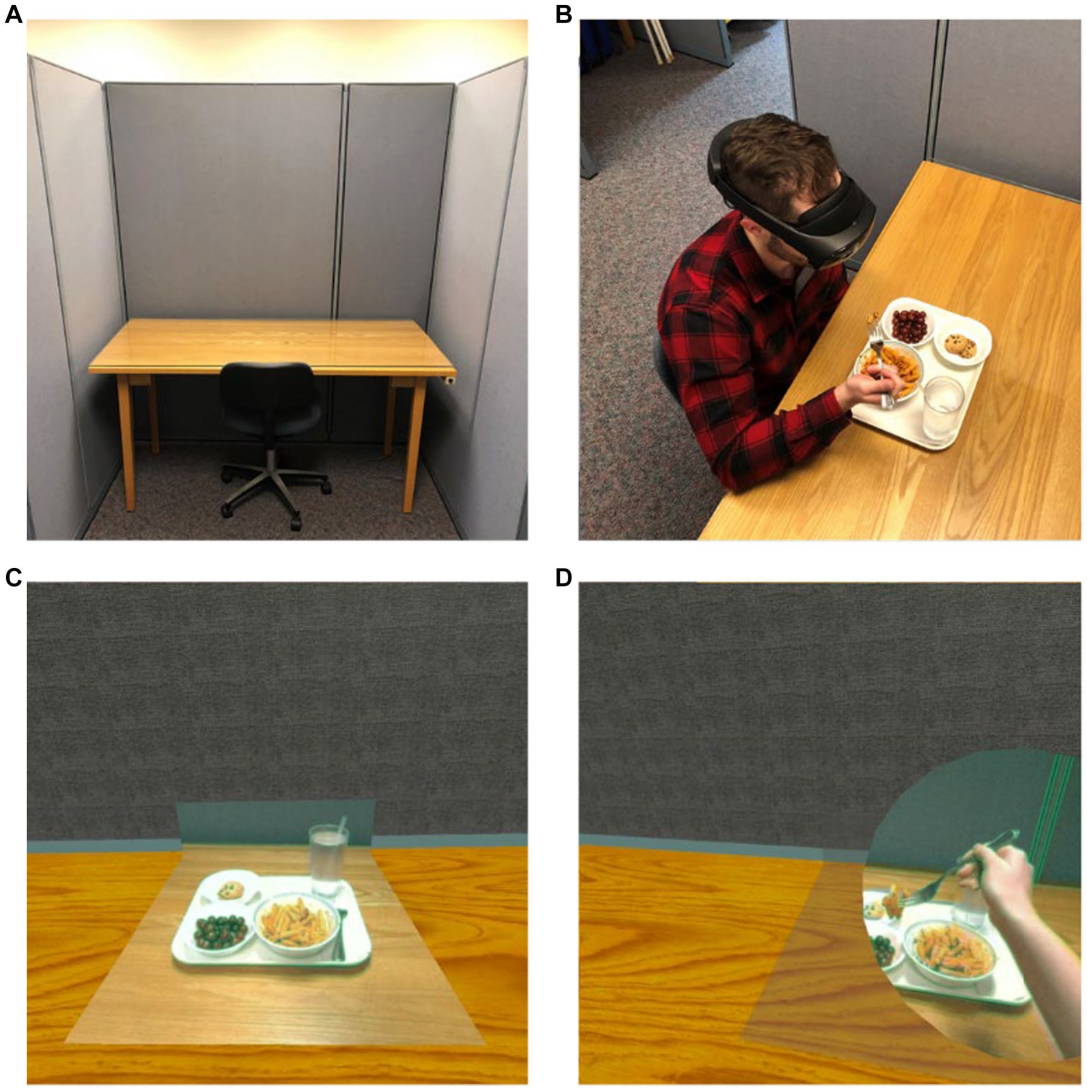
**(A)** Traditional laboratory booth, **(B)** interaction of food in the traditional laboratory booth, **(C)** food in the tabletop passthrough, and **(D)** food in the hand disc passthrough.

For the interaction, the application used the hand tracking feature of the Meta Quest 2 headset. As such, we added two flat passthrough discs at the palm of each of the users’ hands, enabling them to see their real hands and any real-world objects they may be holding such as food or utensils. In order to avoid confusion and visual lensing distortions, a toggle was added which prevents the tabletop passthrough and hand disc passthrough objects from being active at the same time. With this functionality in place, the tabletop passthrough planes remain active until one or both of the hand discs enters a designated area above the tabletop, at which point the passthrough hand discs are activated and the tabletop passthrough planes are disabled. When both passthrough hand discs have left the tabletop area, the passthrough hand discs are disabled and the tabletop passthrough planes are reactivated (Figure 1). This feature allows participants to see and interact with real-life edible food while being immersed within a virtual environment. A visual representation of the specific laboratory booth, food in the laboratory booth, and food in the tabletop passthrough and hand disc passthrough are showcased in Figure 2.

### The intended and potential uses of the application in sensory and consumer science

The application offers a wide range of potential and intended uses for research in sensory and consumer science. The virtual environments provide a controlled and customizable platform for researching factors that influence consumer decision making, such as product interaction, evaluation, and consumer choice. These factors can be assessed within the virtual environments or after exposure to contextual cues within the virtual environments. Additionally, the application allows for the manipulation of sensory cues such as visual presentation, lighting, and background noise, to assess their effect on sensory quality, acceptability, and preferences in a controlled and realistic context. Lastly, the application can be used to assess differences in food intake and eating behavior in a traditional laboratory booth, virtual laboratory booth, and virtual restaurant. This allows researchers to (1) evaluate the validity and feasibility of using virtual contexts for consumption studies, and (2) evaluate the influence of contextual cues on food intake and eating behaviors. Table 1 summarizes the intended and potential uses of the application in sensory and consumer research.

**Table 1:** The intended and potential uses of the application in sensory and consumer research.

Research scenario	Intended and potential uses
Product selection after seeing a context	Simulating virtual environments to study how contextual factors influence consumer decision-making and product selection
Product interaction, evaluation, and selection behavior in a virtual environment	Examining the impact of various factors (e.g., packaging, branding, labeling) on product interaction, evaluation, and consumer choice in a controlled and realistic virtual environment
Assessing sensory quality, acceptability, and preference in virtual environments	Utilizing virtual environments to conduct sensory evaluations, including hedonic testing, discrimination tests, and descriptive analysis, to examine product attributes, consumer preferences, and inform product development and optimization
Product tasting and evaluating in context	Creating immersive environments to explore sensory experiences, including taste perception, aroma, and visual presentation of foods or beverages
Comparing food intake in traditional laboratory booth and virtual laboratory booth	Assessing differences in food intake and eating behavior in traditional laboratory booth and virtual laboratory booth to evaluate the validity and feasibility of using virtual contexts for consumption studies
Comparing food intake and eating behaviors across different virtual environments	Examining differences in food intake behavior among different virtual environments to understand the influence of contextual cues on food intake and eating behaviors

### Recruitment of research experts

While involving consumers in the evaluation of our immersive mixed reality application has its merits, we opted to engage research experts in Nutritional Sciences, Food Science, and Sensory Science for our evaluation for several reasons. Research experts possess specialized knowledge and training in the related domain, allowing them to provide valuable insights and feedback based on their expertise. Their understanding of eating behavior, sensory perception, and food preferences enables them to offer nuanced evaluations of the application’s efficacy and potential impact. Additionally, experts can provide critical input on the scientific validity, usability, and feasibility of the technology, ensuring that the application aligns with established research principles and standards. By involving experts in the early stages of development, we aimed to leverage their expertise to identify and address major usability and validity issues before progressing to larger-scale studies involving consumers and the general population. While consumer perspectives are crucial for assessing real-world applicability and user experiences, our decision to involve experts in this initial evaluation allowed us to benefit from their specialized knowledge and rigorous evaluation criteria, ultimately contributing to the refinement and enhancement of the application.

Eight research experts in Nutritional Sciences, Food Science, and Sensory Science, all of whom are active researchers at our university, were invited to participate in the evaluation based on their background and expertise. A comprehensive email that outlined the nature and objectives of the heuristic evaluation was sent to experts, inviting their participation in the study. This recruitment process identified a sample of six researchers and two professors. The sample size was determined based on prior heuristic evaluations conducted by our group (50, 51) and by others (52, 53). While both heuristic studies had a sample of eight experts, two new researchers were included in heuristic study 2 to introduce new perspectives and ensure a comprehensive evaluation.

### Evaluation procedure for study 1 and study 2

We employed a mixed-methods approach that included think-aloud methodology and a survey as our evaluation methodology. This allowed us to gather both qualitative and quantitative feedback from the experts and provided a comprehensive understanding of their experiences with the application. Experts during the evaluation process engaged in think-aloud techniques and were instructed to verbalize their thoughts, impressions, and recommendations as they interacted with the application. This think-aloud component provided real-time insights, ensured the expert’s feedback was captured accurately, and helped to identify subtle issues that may have otherwise been forgotten once the experience is over. Following the think-aloud phase, we administered a survey that consisted of structured and open-ended questions to gather quantitative and qualitative data. The structured questions provided quantitative data that can be compared across experts and offered a standardized measure of various aspects of the application’s performance. The open-ended questions enabled experts to provide in-depth qualitative feedback. This helped to ensure nuanced insights that may have been missed during the think-aloud portion and through quantitative measures alone were captured. This mixed-methods approach aimed to achieve a comprehensive evaluation of the application, leveraging the strengths of both think-aloud techniques and surveys.

The evaluation process involved two distinct heuristic evaluations: study 1 and study 2. To ensure a comprehensive assessment of the application, we followed an iterative design process, incorporating feedback from research experts at different stages of the application’s development. We implemented a deliberate time interval of several months between the two heuristic studies to facilitate continuous improvement and refinement. The iterative nature of our approach enabled us to gather valuable insights from experts’ initial evaluations and apply those insights to enhance the application’s design and functionality.

Experts followed the same procedure for both study 1 and study 2. Experts participated in the evaluations individually. The expert’s opinions were collected independently and were not influenced by each other. Upon arrival at the laboratory, the experts received an overview of the procedure and objectives of the study. Experts began the evaluation by sampling food in the laboratory booth without the use of the VR headset. Afterwards, the experts were immersed within the application and were allowed to alternate between the various digital environments while eating the foods. The foods presented were pasta dish, grapes, cookies, and a glass of water with a straw. Experts were encouraged to provide detailed feedback during their experience, including their preferences, dislikes, and suggestions for improvement. This dynamic interaction between the research assistant and expert ensured the feedback was captured accurately and facilitated the refinement of the application. Experts had the flexibility to spend as much time as desired immersed within the digital environments. Upon completion of their immersive experience, experts were prompted to complete the heuristic survey and engagement questionnaire (54). The hunger level of the experts was not measured due to the focus of the study being the evaluation of our application rather than on food intake during the session.

### Study 1 – evaluation of the prototype application

#### Primary objective of study 1

Study 1 played a crucial role in our heuristic evaluation by addressing a key aspect of the application’s development: the implementation of passthrough technology. The primary objective was to evaluate the effectiveness of passthrough technology in addressing a key limitation in existing applications – the disconnect between virtual and physical environments. Passthrough has the potential to enhance the application’s realism and immersion by enabling users to see their physical surroundings while immersed in the virtual environments. This feature has the theoretical implication for increasing the plausibility of the scenario and providing a realistic eating experience. Plausibility refers to the illusion that virtual experiences are really happening, which is crucial for participants to fully engage and accept the virtual experience as authentic (55). By seamlessly blending the real and virtual worlds, passthrough allows for novel forms of engagement that would otherwise be difficult to achieve solely within the confines of either realm. However, this is a theoretical argument that requires empirical evidence to definitively support the claims. To address this need and help validate the theoretical argument, we conducted a heuristic evaluation to assess the effectiveness of the passthrough and identify areas of refinement. This early evaluation stage provided valuable insights into the effectiveness of the passthrough feature and identified areas for refinement. For example, nearly all research experts suggested adding social cues such as avatars and background noise to improve our virtual restaurant. This iterative approach ensured that major validity and usability problems were addressed before progressing to larger user studies.

#### Initial test environment

**Figure 3:**
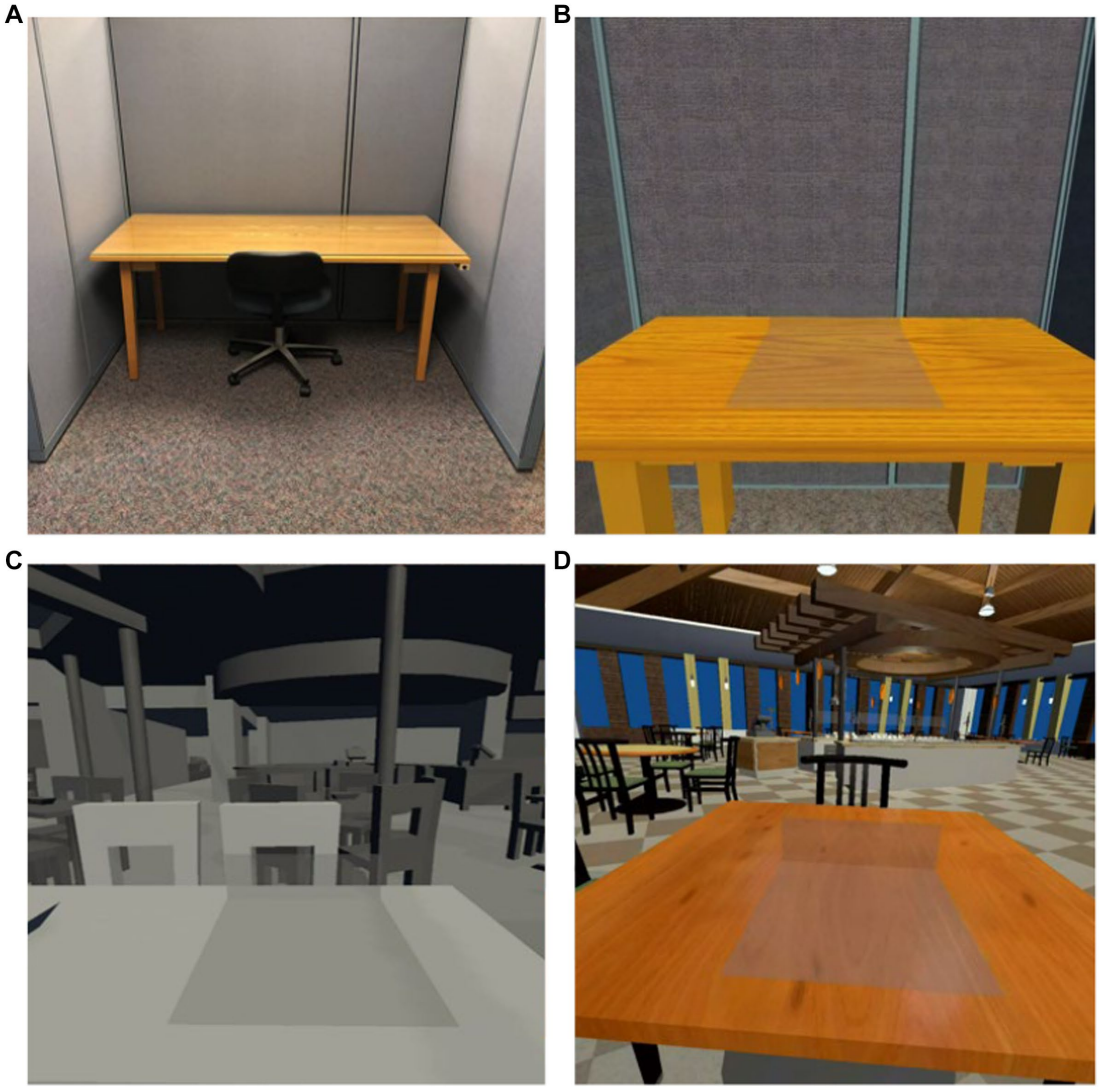
**(A)** Traditional laboratory booth, **(B)** virtual traditional laboratory booth, **(C)** virtual non-textured restaurant, and **(D)** virtual full-textured restaurant.

As the application was created to allow participants to eat while immersed in virtual environments, there was also a need to determine what factors of the virtual environment were most important to eating behavior and sensory research. Therefore, we created 3 “barebone” test environments that were used to elicit feedback from the test users. The three virtual environments included a traditional laboratory booth which was identical to testing booths in the lab, a large non-textured restaurant to elicit responses to sizes, distances, and placement of objects, and a full-textured restaurant to elicit responses on a more realistically modeled environment (Figure 3).

For the first round of developmental feedback a total of 8 experts in eating behavior and sensory science were recruited to evaluate the application. Each expert used the application and was allowed to alternate between the various eating environments while eating the real foods presented. The real foods presented were a pasta dish, grapes, cookies, and a glass of water with a straw. The participants were allowed to speak with the research assistant who was present, and notes were taken by the research assistant during the exploration of the environments.

#### The heuristic survey and engagement questionnaire in study 1

The Heuristic Survey was given to experts following their experience in the application. The experts provided feedback through 27 questions which included a mix of 17 visual analogue (VAS) questions and 10 free-response questions. The VAS questions were categorized into four categories: experience fidelity, interaction fidelity, eating fidelity, and research fidelity. The anchors on each end of the VAS question were used to express the extremes of the feeling such as “not natural at all” to “extremely natural.” The anchors were set to be 0 (worst) to 100 (best). All questions and their corresponding anchor are reported in Table 2.

**Table 2:** Results of the 17 Heuristic Domains in the initial evaluation.

Heuristic	Mean (SD)
*Experience fidelity*	
^a^How natural was your experience in the virtual world (i.e., using your hands and interacting with your food)?	59.50 (30.78)
^d^Was the visual representation of the virtual world true to life?	66.00 (20.53)
^e^How well were you able to find where you are in the virtual environment and return to a known, preset position?	87.25 (17.57)
^f^How much did you feel a sense of being present in the virtual environment?	83.75 (14.85)
*Interaction fidelity*	
^b^Did the virtual hands cause problems with reaching for or grabbing food in a natural manner?	70.37 (29.99)
^c^Were there any observable delays between manipulating the foods in the real world and the virtual interface?	88.25 (28.99)
^b^Was there any delay in rendering during head movement?	88.25 (23.41)
^i^I was able to interact with food items without any restrictions.	72.12 (32.86)
^k^How restrictive was the headset to your eating and drinking?	58.00 (31.73)
^k^How restrictive was the hand interaction in restricting your eating and drinking?	78.37 (23.76)
*Eating fidelity*	
^a^How natural was eating in virtual reality when compared to eating in the real world?	56.87 (25.40)
^g^How comparable was eating in the immersive booth compared to a laboratory booth?	75.62 (19.60)
^g^How comparable was eating in the immersive restaurant compared to a ‘real world’ restaurant?	53.25 (29.29)
^j^The food looked realistic	54.50 (32.91)
^l^The shape of the food in the virtual interface matches the shape of the food in the real world.	86.25 (15.89)
*Research fidelity*	
^h^Eating in a virtual reality experience is an appropriate methodology for measuring eating behavior when compared to traditional methods such as eating in a laboratory booth.	77.5 (11.96)
^h^Eating in a virtual reality experience is an appropriate methodology for measuring eating behavior when compared to eating under real world conditions in a restaurant.	57.87 (25.23)
The anchors were set to be 0 (worst) to 100 (best). ^a^(0) Not at all natural ➔ (100) Extremely natural ^b^(0) Extreme problems ➔ (100) No problems at all ^c^(0) Extreme delays ➔ (100) No delays ^d^(0) Not at all true to life ➔ (100) Extremely true to life ^e^(0) Not at all ➔ (100) Extremely well *^f^*(0) Not at all present ➔ (100) Extremely present ^g^(0) Not at all comparable ➔ (100) Extremely comparable ^h^(0) Not at all appropriate ➔ (100) Extremely appropriate ^i^(0) Not at all able to ➔ (100) Extremely able to ^j^(0) Not at all realistic ➔ (100) Extremely realistic ^k^(0) Extremely restrictive ➔ (100) Not at all restrictive ^l^(0) Not at all matches ➔ (100) Extremely matches	

There was an optional free response field following each VAS question that prompted “please elaborate” to provide specific feedback following each VAS-responses. After the VAS questions, there were 10 free-response questions which asked for feedback on changes that the experts believed would improve the overall experience, what would improve the virtual environment, what foods and meals would be suitable for eating with a head mounted display, and what are the advantages and disadvantages to eating in virtual environments. There was a final optional free response field for any additional information they felt was relevant to the evaluation.

Immersive environments that restore contextual cues often lacking in traditional laboratory testing environments have been shown to improve product discriminability and heighten user engagement leading to more reliable experimental data and higher ecological validity (42, 56). However, engagement is often assessed by conflating questions from the User Engagement Scale (57) and Presence Questionnaire (58). A new questionnaire was developed to measure all dimensions of engagement in the application of food and sensory evaluations (54). The engagement questionnaire measures engagement through active involvement, affective value, and purposeful intent. Active involvement refers to the extent to which individuals focus directly on the task and their ability to not get bored. Affective value refers to any additional interest generated from the sensory evaluation. Purposeful intent refers to the individual’s ability to maintain their level of engagement as a reflection of their perceived relevance of the sensory evaluation. Following the heuristic survey, participants completed the Engagement Questionnaire to assess their engagement with the application (54).

### Study 2 – evaluation of the updated application

#### Primary objective of study 2

**Figure 4:**
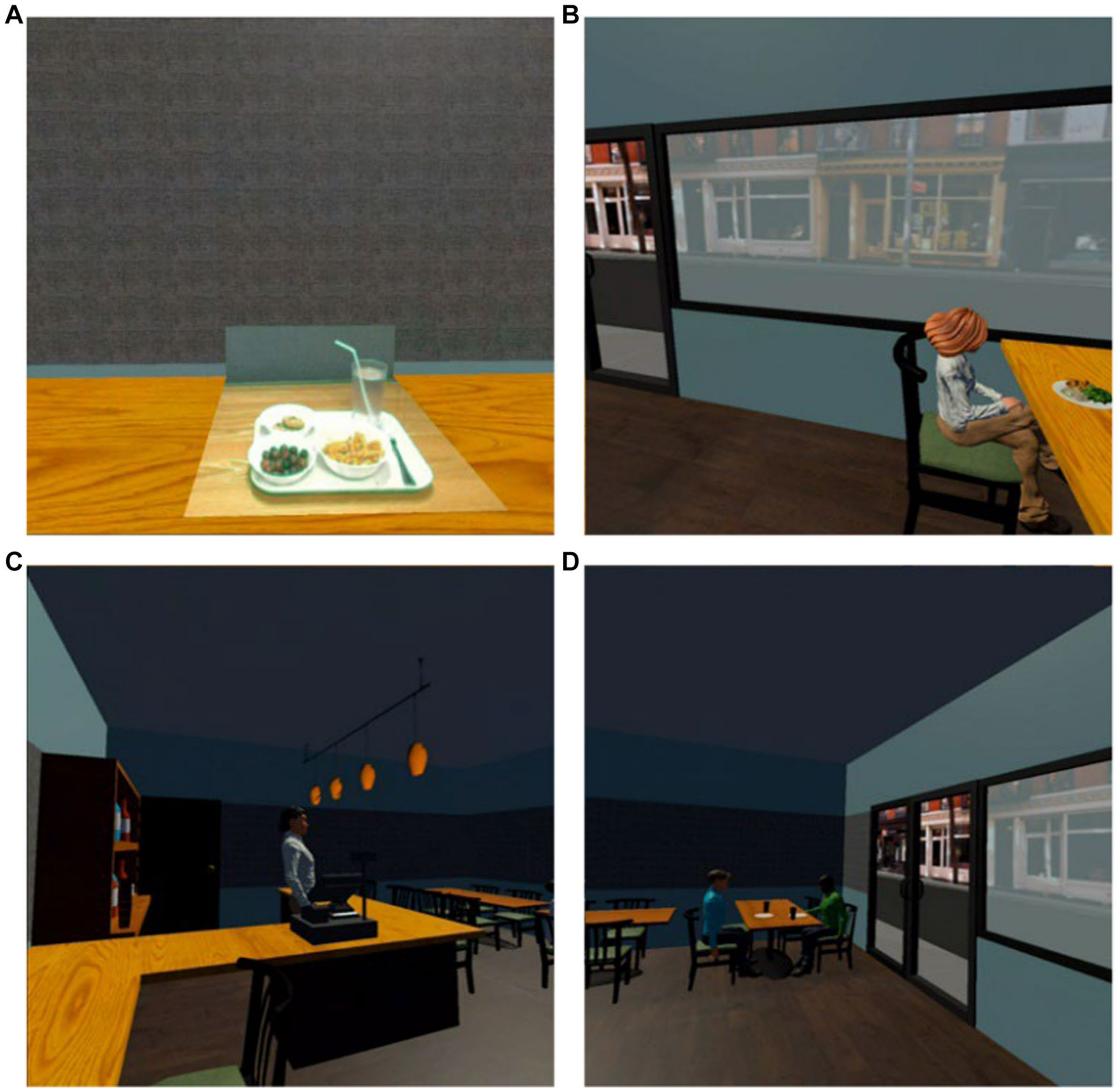
**(A)** Virtual restaurant with foods in the tabletop passthrough, and **(B–D)** virtual restaurant scenery.

Using the feedback from the initial evaluation, the research team redesigned the application by improving the passthrough functionality and creating a new immersive virtual restaurant environment (Figure 4). The new application utilizes full color pass through cameras and therefore the stimuli (i.e., food and utensils) are in color as opposed to black and white. The new virtual restaurant includes improved lighting, virtual avatars, restaurant-specific scenery, and restaurant background noise. The primary objective of study 2 was to seek expert opinion and evaluate the new passthrough feature, as well as the new virtual restaurant environment. This sought to confirm that the updates to our application were aligned with expert expectations, while also assessing the potential utility, ecological validity, and relevance to eating behavior and sensory science research before proceeding to larger-scale studies with a more generalizable sample. The updated version of the application was evaluated by 8 experts in Nutritional Sciences, Food Science, and Sensory Science. The foods presented during this trial were identical to the initial evaluation. Each expert followed the same protocol from the initial evaluation. This evaluation stage provided insights to determine the readiness of the technology for further testing and validation in larger-scale studies involving a more general population.

#### The heuristic survey and engagement questionnaire in study 2

A new Heuristic Survey was given to experts following their experience with the application. The new Heuristic Survey comprised of similar questions; however, wording was modified on some pre-existing questions to reduce ambiguity. Additionally, questions that centered around highly scored features that did not warrant a developmental change were removed. The Heuristic Survey was given to experts following their experience in the application. The experts provided feedback through 25 questions which included a mix of 14 visual analogue (VAS) questions and 11 free-response questions. The VAS questions were categorized into four categories: experience fidelity, interaction fidelity, eating fidelity, and research fidelity. The anchors on each end of the VAS question were used to express the extremes of the feeling such as “not natural at all” to “extremely natural.” The anchors were set to be 0 (worst) to 100 (best). All questions and their corresponding anchor are reported in Table 3.

**Table 3:** Results of the 13 Heuristic Domains in the final evaluation.

Heuristic	Mean (SD)
*Experience fidelity*	
^a^How natural was your experience in the virtual restaurant (i.e., using your hands and interacting with your food)?	69.62 (15.20)
^c^Was the visual representation of the virtual restaurant true to life?	67.37 (11.93)
^d^How much did you feel a sense of being present in the virtual environment?	68.87 (16.18)
*Interaction fidelity*	
^b^Did the virtual hands cause problems with reaching for or grabbing food in a natural manner?	78.62 (18.33)
^b^Was there any delay in rendering during head movement?	86.87 (22.04)
^g^I was able to interact with food items without any restrictions.	82.50 (19.93)
^i^How restrictive was the headset to your eating and drinking?	75.37 (25.03)
^i^How restrictive was the hand interaction in restricting your eating and drinking?	78.75 (26.83)
*Eating fidelity*	
^a^How natural was eating in the virtual restaurant when compared to eating in the real world?	62.75 (19.72)
^e^How comparable was eating in the immersive restaurant compared to a ‘real world’ restaurant?	58.62 (15.82)
^h^The food in the pass-through video looked as expected.	67.25 (21.17)
*Research fidelity*	
^f^Eating in a virtual restaurant is an appropriate methodology for measuring eating behavior when compared to traditional methods such as eating in a laboratory booth.	71.50 (14.81)
^f^Eating in a virtual restaurant provides contextual cues similar to those encountered when eating in a restaurant.	65.50 (24.45)
The anchors were set to be 0 (worst) to 100 (best). ^a^(0) Not at all natural ➔ (100) Extremely natural ^b^(0) Extreme problems ➔ (100) No problems at all ^c^(0) Not at all true to life ➔ (100) Extremely true to life ^d^(0) Not at all present ➔ (100) Extremely present ^e^(0) Not at all comparable ➔ (100) Extremely comparable ^f^(0) Not at all appropriate ➔ (100) Extremely appropriate ^g^(0) Not at all able to ➔ (100) Extremely able to ^h^(0) Not at all realistic ➔ (100) Extremely realistic ^i^(0) Extremely restrictive ➔ (100) Not at all restrictive	

There was an optional free response field following each VAS question that prompted “please elaborate” to provide specific feedback following each VAS-responses. After all the VAS questions, there were 11 free-response questions which asked for feedback on changes that the experts believed would improve the overall experience, what would improve the virtual environment, what foods and meals would be suitable for eating with a head mounted display, and what are the advantages and disadvantages to eating in virtual environments. There was a final optional free response field for any additional information they felt was relevant to the evaluation. Following the heuristic survey, participants completed the Engagement Questionnaire to assess their engagement with the application (54).

## Results

### Study 1 – results

**Figure 5:**
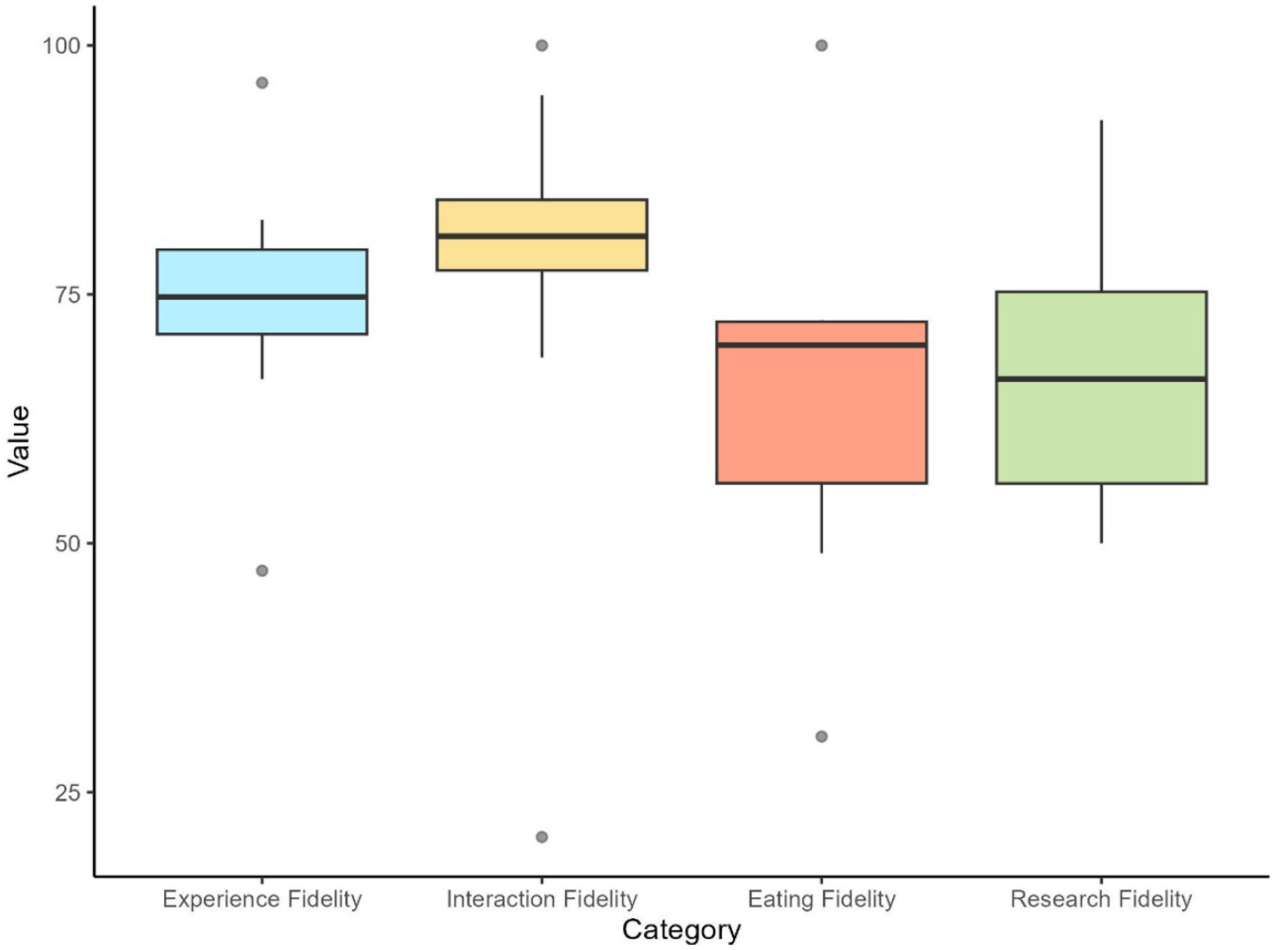
Boxplots illustrate the distribution of mean expert ratings for the four heuristic categories in the initial evaluation. The mean score for each expert within each category was calculated by averaging the scores of all questions belonging to that category for each individual expert.

The specific results for each VAS question and heuristic category are reported in Table 2. Boxplots illustrating the distribution of mean expert ratings for the four heuristic categories are reported in Figure 5. The specific free-response question, general theme of the answers, and a highlighted response from one expert are reported in Table 4. All responses to the free-response questions can be found in Supplementary Table 1. The application’s engagement results scored a 5.50 on active involvement, 5.53 on purposeful intent, and 5.54 on affective value on a 7-point scale. The results of all dimensions of engagement are reported in Table 5.

**Table 4:** Results of the free-response questions in the initial evaluation.

Question	General theme	Highlighted response
Q1. If you were manipulating a virtual environment for a study, what would you add? What would you remove?	Add social elements such as people or avatars.	“People at other tables. Background noise was great!”
Q2. If you were using this technology for a study, what food(s) and/or meal(s) would you serve?	Foods that are easily manipulated and would avoid hitting the headset.	“Finger foods to avoid issues of the fork/utensils hitting the headset.”
Q3. If any, what are the advantages of eating in virtual reality compared to eating in a booth in the laboratory?	Provides researchers with the ability to manipulate and control various environmental and food-related cues.	“We are concerned about how people’s behaviors in lab booth translate into real-life behaviors. VR could be a way to maintain experimental control and at the same time evoke that real-life context.”
Q4. If any, what are the disadvantages of eating in virtual reality compared to eating in a booth in the laboratory?	Wearing a headset while eating and virtual environments may be novel and act as a distractor from food.	“Could cause nausea in some individuals which may decrease appetite. Some individuals may be so intrigued by the technology that they are less interested in eating.”
Q5. To what extent do you think eating in virtual reality can replace current methods for researching eating behavior such as eating in a laboratory booth?	The current technology cannot fully replace current methodologies but can be used to assess influences that otherwise would not be possible in a traditional laboratory setting.	“I think that virtual reality can add to the field and allow for studies that otherwise would not be possible, however I do not think it can completely replace real world research.”
Q6. What would you change about the virtual reality experience to improve upon the experience?	Improvements in the technology that would allow for lighter and less restrictive headsets, improved graphics, and viewing the food in color.	“Improving the graphics and allowing for the food to appear in color would be helpful.”
Q7. What benefits, if any, do you see in changing environments for eating behavior research?	Enables researchers to assess environmental influences and individual factors while maintaining adequate control of other variables.	“We know that context can have a significant influence on eating behavior and so changing the environment allows researchers to manipulate and explore different contexts.”
Q8. Did the look of the food in the interface affect the taste of the food?	No.	No.
Q9. Were there any difficulties with interacting with food items?	Overall, no difficulties.	“I did not experience any difficulties.”
Q10. Any additional comments.	NA	NA

**Table 5:** Results of the Engagement Questionnaire in the initial evaluation.

Item	Mean (SD)
*Active involvement*	*5.50 (1.93)*
Lost interest	5.37 (2.13)
Distracted	4.50 (2.20)
Zoning out	6.62 (0.51)
*Purposeful intent*	*5.53 (1.34)*
Attention	5.50 (1.69)
Significant	5.62 (1.06)
Meaningful	5.37 (1.50)
Dedicated	5.62 (1.30)
*Affective value*	*5.54 (1.66)*
Motivation	5.37 (1.84)
Captivating	6.00 (0.75)
Enjoyment	5.25 (2.18)

Participants rated their level of agreement to the questions using a 7-point Likert scale from strongly disagree (1) to strongly agree (7). All items in the active involvement are reverse-coded due to the negatively worded question format.

### Study 1 – qualitative results

The two iterations of the developed application were evaluated by expert researchers in the fields of Nutritional Sciences, Food Science, and Sensory Science based on the potential utility, ecological validity, and relevance to eating behavior and sensory-related research. Study 1 revealed that our initial digital restaurant environment lacked social elements. Nearly all researchers suggested adding social elements such as people or background noise in response to the question “if you were manipulating a virtual environment for a study, what would you add?” A researcher noted “I would add background noise (e.g., background chatter) and people to make it reflect the real world.” Therefore, in the second iteration of the application we added background noise and avatars in several locations throughout our new digital restaurant environment. While this general comment seemed to be resolved in the second version of the application, participants suggested continuing to improve décor. This demonstrates that our experts see the great value and flexibility that virtual environments bring in modifying/improving the aesthetics of the experience. Additionally, virtual environments open the door for numerous research opportunities to understand the effects of manipulating different visual cues within the environment and their effect on eating behavior.

The research experts in study 1 noted that the constraints of the current technology limited their ability to view the food in full color and subsequently hindered the realism of their experience. This was highlighted in many questions in the eating fidelity and research fidelity category such as “how natural was eating in virtual reality when compared to eating in the real world?” Additionally, the theme of the free-response question “What would you change about the virtual reality experience to improve upon the experience,” consisted of improvements in technology that would facilitate less restrictive headsets, improved graphics, and viewing the food in color. This was highlighted by the quote “Improving the graphics and allowing for the food to appear in color would be helpful.” The new application is run on the Meta Quest Pro with improved processing power, graphics, and full color passthrough and this issue appeared to be resolved when considering comments from Study 2.

### Study 2 – results

**Figure 6:**
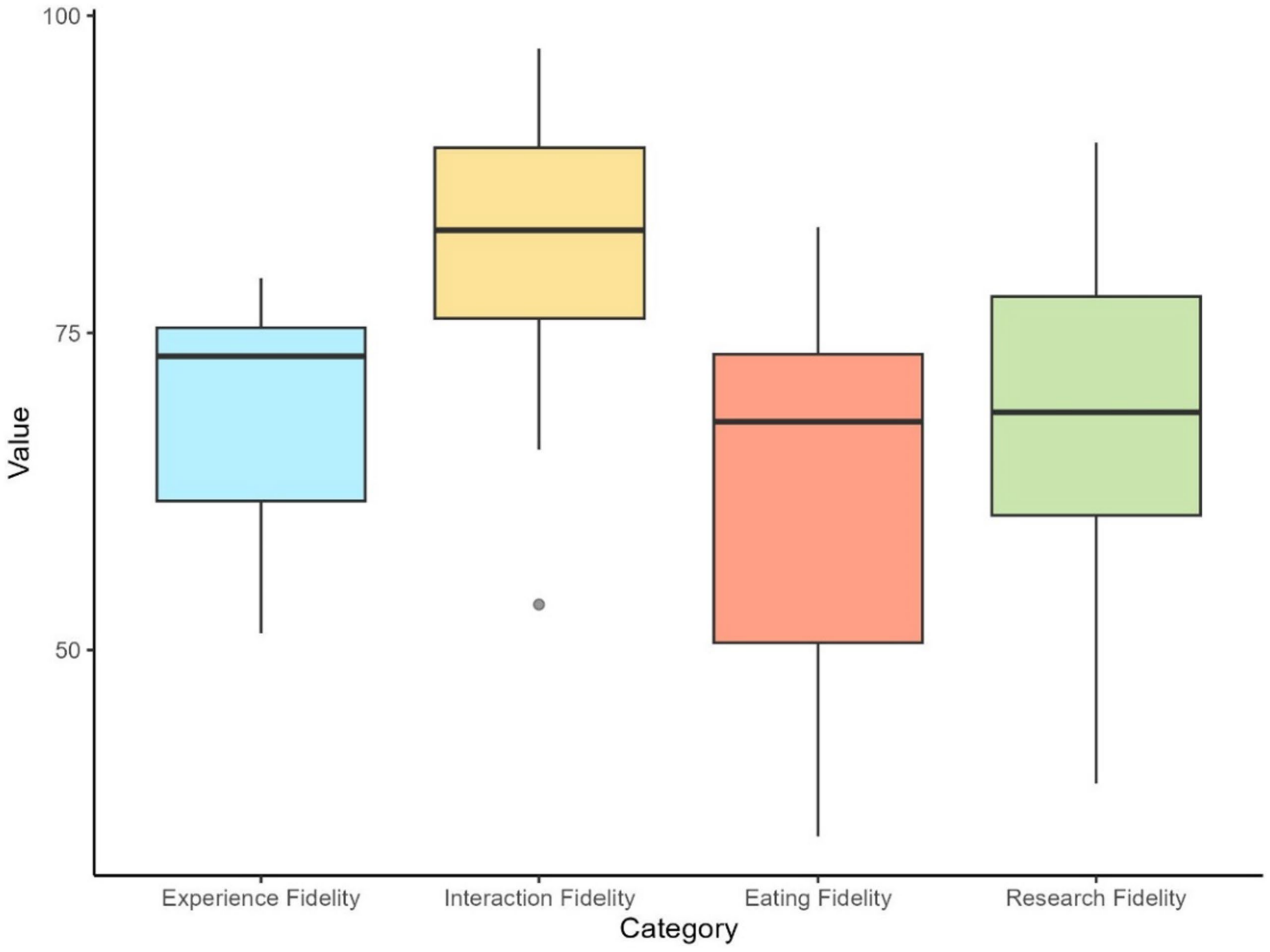
Boxplots illustrate the distribution of mean expert ratings for the four heuristic categories in the final evaluation. The mean score for each expert within each category was calculated by averaging the scores of all questions belonging to that category for each individual expert.

The specific results for each VAS question and heuristic category are reported in Table 3. Boxplots illustrating the distribution of mean expert ratings for the four heuristic categories are reported in Figure 6. The free-response question, general theme of the answers, and a highlighted response from one expert are reported in Table 6. All responses to the free-response questions can be found in Supplementary Table 2. The application’s engagement results scored a 5.66 on active involvement, 5.90 on purposeful intent, and 5.70 on affective value on a 7-point scale. The results of all dimensions of engagement are reported in Table 7.

**Table 6:** Results of the free-response questions in the final evaluation.

Question	General theme	Highlighted response
Q1. If you were manipulating a virtual environment (such as a virtual restaurant) for a study, what would you add? What would you remove?	Add décor and background noise that would be typical of a restaurant.	“I’d add ambient sounds (in accordance with how busy the place seemed due to the number of patrons), and some decoration on the walls of the restaurant.”
Q2. If you were using this technology for a study, what food(s) and/or meal(s) would you serve?	Serve a meal that is easy to eat and not messy while also fits the simulated eating environment.	“Depends on the environment. The café looks like a place that would have sandwiches, soup, salad, and baked goods. I think the type of environment dictates the food served. The food served would need to be believable as a something that would be served in a restaurant.”
Q3. If any, what are the advantages of eating in virtual reality compared to eating in a booth in the laboratory?	Provides researchers with the ability to manipulate and control various environmental and food-related cues.	“The ability to freely manipulate context and food cues is a huge benefit that practically is difficult to achieve in a lab booth.”
Q4. If any, what are the disadvantages of eating in virtual reality compared to eating in a booth in the laboratory?	There may be novelty effects of virtual environments as well as the discomfort of eating while wearing a headset.	“It is not completely natural - there may be novelty effects or more distractions”
Q5. To what extent do you think eating in virtual reality can replace current methods for researching eating behavior such as eating in a laboratory booth?	Virtual reality is a strong methodology for evaluating environmental cues but cannot replace a traditional laboratory booth for food focused manipulations.	“I believe they might complement each other. Sensory booths are great for removing all possible variables and creating a very sterile experience, whereas the virtual reality will be great for adding more environments and food cues, and improving ecological validity”
Q6. What would you change about the virtual reality experience to improve upon the experience?	Add background noise.	“Add more details to bring more life to the environment. Developments in tech will also help improve the limitations of the real-world window.”
Q7. What benefits, if any, do you see in changing environments for eating behavior research?	Researching environmental influences of eating behavior.	“We know that different environments and cues can influence eating behavior. VR allows for more exploration of this.”
Q8. Did the look of the food in the interface affect the taste of the food?	No but some noted a small difference between the colors of the food in the real world and in VR.	“Because of the color difference between the interface and the real-world, there was increased uncertainty about what to expect for the first bite. But after that, the taste was not impacted.”
Q9. Were there any difficulties with interacting with food items?	No, but some noted utensils and a cup may evoke issues with participants hitting the headset.	“No, besides the water situation.”
Q10. Did you experience motion sickness or any changes in nausea during your experience in VR?	No.	“None at all.”
Q11. Any additional comments.	Technology is improving.	“This is impressive technology with very promising applications!”

**Table 7:** Results of the Engagement Questionnaire in the final evaluation.

Item	Mean (SD)
*Active involvement*	*5.66 (1.37)*
Lost Interest	5.87 (0.99)
Distracted	5.00 (2.00)
Zoning Out	6.12 (0.64)
*Purposeful intent*	*5.90 (1.25)*
Attention	6.37 (1.06)
Significant	5.25 (1.58)
Meaningful	5.75 (1.16)
Dedicated	6.25 (1.03)
*Affective value*	*5.70 (1.23)*
Motivation	5.62 (1.40)
Captivating	5.50 (1.30)
Enjoyment	6.00 (1.06)

Participants rated their level of agreement to the questions using a 7-point Likert scale from strongly disagree (1) to strongly agree (7). All items in the active involvement are reverse-coded due to the negatively worded question format.

### Study 2 – qualitative results

The findings and feedback from study 1 facilitated several key modifications of the application that were implemented for study 2. The changes included improvements in the lighting, such as a brighter ambiance and decorative ceiling lighting, aimed at creating a more realistic restaurant environment. The social elements of the restaurant were improved by including virtual avatars throughout the environment. Lastly, the passthrough feature was updated with improved functionality and to provide a full-color display and therefore the stimuli (i.e., food and utensils) are in full-color as opposed to black and white. These changes aimed to enhance the utility, ecological validity, and relevance to eating behavior and sensory-related research.

The research experts in study 2 noted that food served in MR studies should be befitting of the simulated eating environment. This was highlighted by the quote “It would vary according with the environment being simulated for improved ecological validity. The types of food served in an à la carte restaurant are different from those served at a café and a cafeteria, for example.” Another comment from study 2 was to increase the background noise present in the environment. However, others were concerned that increasing the background noise would be too distracting. This raises an interesting potential research avenue as the level and complexity of the background/ambient sounds can be easily and tightly manipulated in future studies. In light of this, sound could be considered as another environmental cue that can be manipulated and explored in future studies related to understanding the eating behavior of users in different settings.

## Discussion

The present application is a technology-based methodological advancement that is designed to modify eating environments and improve the ecological validity of traditional laboratory-assessed eating behavior. The application permits researchers the ability to manipulate and control environmental and food-related cues while maintaining control of confounding variables that are otherwise difficult to control in other testing environments. However, further studies involving larger and more diverse samples, including end users and consumers, are necessary to validate the technology. Additionally, the application is the first to our knowledge to allow participants to view and eat food while immersed in a digital environment.

Our heuristic questions related to research fidelity highlight some important aspects of the virtual eating restaurant. Specifically, we aimed to address the following questions: (1) “Eating in a virtual restaurant is an appropriate methodology for measuring eating behavior when compared to traditional methods such as eating in a laboratory booth.” This question revealed that experts found the virtual restaurant to be a potentially suitable approach for studying eating behavior when compared to the laboratory booth, although further validation studies with larger and more diverse samples are needed. (2) “Eating in a virtual restaurant provides contextual cues similar to those encountered when eating in a restaurant.” This question revealed that experts recognized the virtual restaurant environment as having contextual cues but acknowledged further improvements are necessary to replicate an authentic restaurant experience. This suggests that our design shows promise in improving the ecological validity of the laboratory through modifying the eating environment. However, the lower scores in relation to the virtual ecologically relevant setting (i.e., virtual restaurant) suggest that the digital environment does not fully replicate a restaurant experience. This is supported by comments such as, “This may be a good in-between to measuring ‘typical’ eating environments with more controls than would be possible normally. Researchers should note that the use of a virtual restaurant will not likely fully replace field studies but offer a unique way to increase experimental control while potentially increasing the ecological validity of findings. Virtual environments also provide a way to rapidly test potential research paradigms prior to engaging in more costly field studies.

In addition to the heuristic questionnaire experts also completed the Engagement Questionnaire in both studies. The scores between the two studies show marginal improvement in all categories, suggesting that the current application is more engaging in all categories measured as compared to our previous version. In the context of other literature, Zandstra et al., found participants’ engagement to be significantly higher within an immersive simulated café and a real café when compared to a laboratory setting (59). The engagement in their immersive simulated café was an intermediate between the laboratory setting and real café. This is similar to the results of our application improving the ecological validity of the laboratory but not fully replicating a restaurant experience.

The heuristic evaluations of our application have provided valuable insights and recommendations for future research in this domain. From a developmental insight, researchers should match the color of the digital booth to their laboratory booth to ensure color consistency through the hand passthrough discs. This enhances the overall visual experience and improves the participant’s sense of immersion. The addition of social elements, such as people or avatars, in the virtual restaurant environment was found to enhance the representation of a typical restaurant experience. However, it is important to consider participant preferences, as some experts noted that participants may find constant eye contact from avatars uncomfortable. To address this, our updated restaurant deliberately positioned avatars to be engaged in their own activities such as eating, talking to each other, and facing away from participants. Another insight learned is the careful selection of foods and beverages to avoid potential mess or damage to the headset equipment. Experts advised choosing foods that are easy to eat and minimize the risk of spills, such as fingers foods and beverages with a lid and straw. Lastly, researchers should be mindful that immersing participants in digital environments may introduce a novelty effect that may alter food consumption. Researchers can mitigate this by allowing participants an initial or thorough immersion experience before eating within the virtual environment. These insights and recommendations contribute to the advancement of utilizing this new methodology in eating behavior and sensory-related research.

The passthrough technology implemented in our application provides a seamless integration of virtual and physical realms and represents an advancement in creating a more immersive virtual eating experience. This has notable implications for the study of eating behavior, sensory science, and consumer science as the feature allows for a more accurate representation of real-world contexts within a controlled laboratory setting. While immersive technologies have shown to improve testing environments compared to traditional laboratory environments, the previous limitation of not being able to visually see and interact with the food while immersed in the virtual environment has hindered the authenticity of these simulations (48, 49). For example, previous research has utilized a desk raiser to raise beverages to participants as opposed to the participants being able to see and interact with the beverages (49). However, the integration of passthrough technology opens new avenues for enhancing existing methodologies. Passthrough technology has been proven valuable in other fields by improving simulations for designing and validating the safety of novel driver assistance features (60). Inspired by this success, we believe that the passthrough feature can be similarly utilized to advance eating behavior, sensory science, and consumer science research. The integration of passthrough technology in our application addresses some of the limitations of previous methodologies and has the potential to enable more authentic and ecologically valid research in the fields of eating behavior, sensory science, and consumer science.

Extended Reality technologies have been used to improve the environmental attributes of the laboratory beyond restaurant environments. For example, dark chocolate in a virtual live concert was found to significantly improve consumers’ hedonic response and emotions when compared to a sensory booth control and a sightseeing tour (61). Additionally, the usage of new digital real-world environments such as VR buffets and VR supermarkets provide insight into consumer responses (26, 62). Food selection within an immersive VR buffet was validated by correlating the selection of food in a VR buffet and a real-world buffet. There was a significant and positive correlation with food weight, energy content, and macronutrient distribution between conditions (26). These results indicate that immersive technologies can be used to measure real-world behavior within the laboratory setting. Similarly, the integration of the passthrough feature in the current studies provides a seamless integration of virtual and physical realms that helps to bridge the gap between real-world contexts within a controlled laboratory setting. By harnessing the power of newer generation iVR head mounted displays that mix virtual and real objects in the same immersive experience; we aim to replicate authentic real-world environments and provide users natural and realistic interactions with physical stimuli that engage other senses such as taste and smell.

### Future directions

Although the results of the present application indicate a technology-based methodological advancement, the sample of participants are a small number of research experts with expertise in nutrition and sensory research. Therefore, a larger, more generalized sample is needed to further validate the digital environment. Additionally, research is warranted to determine whether eating behavior is similar in digital restaurant environments and real-world restaurant environments. The present application can be used to determine potential differences between a digital restaurant and a real-world restaurant.

Although there is a body of literature that indicates using low-immersive virtual reality screens such as television or a smartphone while eating increases energy intake, there is a dearth of literature evaluating whether highly immersive mixed reality (such as a head-mounted display) experiences increase energy intake or changes eating behavior (63). The present application can be used to evaluate differences in eating behavior by assessing differences in food consumption in a traditional sensory booth and a highly immersive virtual sensory booth, as well as a virtual restaurant. Additionally, the present application can be modified to assess the effect of various environmental aspects on eating and sensory-related behavior. For example, researchers are able to specifically isolate variable(s) of interest and determine their effect on eating outcomes. Lastly, the present application can be used to assess sensory differences between visual cues and sensory-related outcomes by distorting the visual cues of food within the immersive environment.

Immersive VR has become increasingly prevalent in the usage of nutrition education (32). For example, nutrition education applications have been designed and shown to improve portion size control and food energy density knowledge (33). However, many studies conducted have focused on the design of the application and indicated potential, but research on the impact of immersive virtual nutrition education applications is limited (32). The present MR application can be modified to include an immersive digital coach and provide feedback while participants consume real foods in various digital environments. This may increase the evidence for impact and help identify specific interventions for an individual rather than designing a one size fits all nutrition education program.

Screen time in the absence of food advertising has been associated with increased dietary intake due to the interference of physiological food regulation, screens being a conditioned cue to eat, and distraction (6). Technology companies have put a heavy emphasis on creating interactive digital environments (e.g., The Metaverse) that merge physical reality with digital reality and allow for multisensory interactions within virtual environments such as interactions with digital people (64). Interactive digital environments may create a new social facilitation as individuals will be able to eat socially in a unique manner as interaction will be occurring through interactive digital environments (65). Social facilitation in eating behavior refers to the phenomenon in which people eat more with others than when eating alone (7). We suspect immersive interactive digital environments may alter the eating environment through disinhibited/distracted eating via digital stimuli.

### Limitations

The present application drew strengths on a novel methodological approach that allows participants to view stimuli while being immersed in digital environments, yet it is not without limitations. The major limitation of this study is that the heuristic evaluation of the application is from a small number of research experts who specialize in nutrition and sensory research and therefore these results cannot be extrapolated or used to fully validate the digital environment. Additionally, the pasta dish, grapes and cookies may not be entirely congruent and realistic within the virtual restaurant environment. Lastly, there is a possibility of the previous use of VR headsets influencing the response to virtual environments. We did not determine whether the experts had previously used VR headsets or had any preconceived notions about the use of immersive virtual technology in measuring eating behaviors.

## Conclusion

Although immersive technologies are a nascent technology in food-related research, the results of this evaluation suggest mixed reality has the potential to be used as a methodological tool to improve the ecological validity of traditional laboratory eating environments but may not serve as a complete replacement for real world conditions.

## Data availability statement

The original contributions presented in the study are included in the article/Supplementary material, further inquiries can be directed to the corresponding author.

## Ethics statement

Ethical review and approval was not required for the study of human participants in accordance with the local legislation and institutional requirements.

## Author contributions

JL, PS, and TM were responsible for the design. BM was responsible for software development. JL and TM were responsible for manuscript preparation. PS, BM, and CS were responsible for manuscript editing. All authors contributed to the article and approved the submitted version.

## Conflict of interest

The authors declare that the research was conducted in the absence of any commercial or financial relationships that could be construed as a potential conflict of interest.

## Publisher’s note

All claims expressed in this article are solely those of the authors and do not necessarily represent those of their affiliated organizations, or those of the publisher, the editors and the reviewers. Any product that may be evaluated in this article, or claim that may be made by its manufacturer, is not guaranteed or endorsed by the publisher.

## Footnotes

^1^https://www.microsoft.com/en-us/hololens

^2^https://www.meta.com/quest/quest-pro/
