# Supplementary material for: The development of an immersive mixed-reality application to improve the ecological validity of eating and sensory behavior research
Source: Front Nutr. 2023 Jul 19;10:1170311. doi: 10.3389/fnut.2023.1170311 (PMC10395832; doi:10.3389/fnut.2023.1170311)
Supplement: Supplementary file 1 [file Data_Sheet_1.docx]

**Appendix A**

***Heuristic Evaluation Questionnaire***

Instructions: Please answer the following questions on a scale from 0-100 (0 being worst, 100 being best) for each of the following questions. There will be a free response box after each question to further explain or comment. Please answer “NA” if you do not have an answer as each question requires a response to complete the survey.

Q1. How natural was eating in the virtual restaurant when compared to eating in the real world?

Please elaborate.

Q2. How natural was your experience in the virtual restaurant (i.e. using your hands and interacting with your food)?

Please elaborate.

Q3. Did the virtual hands cause problems with reaching for or grabbing food in a natural manner?

Please elaborate.

Q4. Was the visual representation of the virtual restaurant true to life?

Please elaborate.

Q5. Was there any delay in rendering during head movement?

Please elaborate.

Q6. How much did you feel a sense of being present in the virtual restaurant?

Please elaborate.

Q7. Eating in a virtual restaurant is an appropriate methodology for measuring eating behavior when compared to traditional methods such as eating in a laboratory booth.

Please elaborate.

Q8. Eating in a virtual restaurant provides contextual cues similar to those encountered when eating in a restaurant.

Please elaborate.

Q9. I was able to interact with food items without any restrictions.

Please elaborate.

Q10. The food in the pass-through video looked as expected.

Please elaborate.

Q11. How restrictive was the headset to your eating and drinking?

Please elaborate.

Q12. How restrictive was the hand interaction in restricting your eating and drinking?

Please elaborate.

Q13. How comparable was eating in the immersive restaurant compared to a "real world" restaurant?

Please elaborate.

Q14. If you were manipulating a virtual environment (such as a virtual restaurant) for a study, what would you add? What would you remove?

Q15. If you were using this technology for a study, what food(s) and/or meal(s) would you serve?

Q16. If any, what are the advantages of eating in virtual reality compared to eating in a booth in the laboratory?

Q17. If any, what are the disadvantages of eating in virtual reality compared to eating in a booth in the laboratory?

Q18. To what extent do you think eating in virtual reality can replace current methods for researching eating behavior such as eating in a laboratory booth?

Q19. What would you change about the virtual reality experience to improve upon the experience?

Q20. What benefits, if any, do you see in changing environments for eating behavior research?

Q21. Did the look of the food in the interface affect the taste of the food?

Q22. Were there any difficulties with interacting with food items?

Q23. Did you experience motion sickness or any changes in nausea during your experience in VR?

Q24. Any additional comments.

**Appendix B**

| **Open Ended Responses to the Heuristics Questionnaire** | |
| --- | --- |
| **Respondent** | **Response** |
| **Q1.** How natural was eating in the virtual restaurant when compared to eating in the real world? | |
| **1** | feels like you're actually in the environment. It's animated- but you feel like youre inside it- not just a spectator. |
| **2** | the virtual restaurant environment without any sound was unnatural; see additional comments about the representation of the restaurant being true to life below |
| **3** | It was clear that efforts had been made to make it close to a real restaurant environment (i.e. other people eating, restaurant workers, a window showing a world that exists outside of the restaurant). It wasn't completely natural since the graphics were clearly simulated and the view in the real world window was dull in color and slightly blurry. |
| **4** | Although I was able to tell where the real world stopped and the VR began, that didn't prevent me from having a pretty realistic eating experience. |
| **5** | VR does well to replicate a digital version of a cafe. The avatars add to the realistic environment of the cafe. However, it still feels very virtual. |
| **6** | pretty natural but it's still a virtual environment. The upgrades (people, environment, etc.) made it much more enjoyable |
| **7** | The VR did a good job at mimicking a real restaurant, despite the cartoony visual of the patrons and staff. The table being the same as the one in the lab adds a nice touch. The field of view disappearing and the displacement of color from shapes when moving the hands is the biggest factor that detracts from the realism of the experience. |
| **8** | I liked the set up of the cafe, it felt realistic. |
| **Q2.** How natural was your experience in the virtual restaurant (i.e. using your hands and interacting with your food)? | |
| **1** | only issue is I can only see whats behind my hands , so slightly unnatural. |
| **2** | when the food tray was in the square on the table (such that it was always visible), eating felt pretty natural. it was less natural when the food was not in the square and only visible via the pass-through with the hand, since the food was disappear when the hand was pulled away |
| **3** | The food was visible in the window and the positioning in my vision was accurate (i.e. everything was where I was expecting it to be when I reached for it). The headset wasn't really in the way (however I didn't try to drink which might have been more of an issue). |
| **4** | It worked pretty well for the most part! Grabbing the cup/straw and finger food were no problem, but the fork was a bit harder to navigate. Once I got used to finding my mouth with the fork, it wasn't a big issue. |
| **5** | For the most part moving my hands and picking up the food felt natural, but the way the images change as my hands move felt less natural. |
| **6** | Interaction with food using my hands is pretty seamless; the circles around my hands sometimes don't overlap and make it difficult to see the food. Generally, pretty good though |
| **7** | The way the size and color of foods get distorted when moving the hands makes it feel like my hands are my eyes, thus changing the way I interact with objects around me |
| **8** | New headset was a big improvement. Cafe and people were good. |
| **Q3.** Did the virtual hands cause problems with reaching for or grabbing food in a natural manner? | |
| **1** | no issues. |
| **2** | NA |
| **3** | the virtual hands weren't an issue since whenever I put my hands out to grab I saw my real hands in the window. |
| **4** | I didn't perceive any issues with the virtual hands. As I mentioned, the only struggle was finding my mouth with the fork, but that was more reorienting my spatial awareness than trouble with the hands. |
| **5** | I don't think it changed how I moved my hands, but the image changing was visual distracting. |
| **6** | see above |
| **7** | Reaching and grabbing was a little odd due to feeling like my hand movements and my vision were tied to each other, but it was something I could overcome and eat without making a mess |
| **8** | The telescoping artefact when bringing food to face still needs a little work. |
| **Q4.** Was the visual representation of the virtual restaurant true to life? | |
| **1** | yes, except for the animation it was very similiar to real life. |
| **2** | the virtual restaurant environment without any sound was unnatural; the virtual person sitting next to me but not acknowledging me in any way and not interacting with her food was unnatural. the body language of the people sitting / chatting behind me felt realistic, as well as the guy behind the counter. |
| **3** | I liked the detail of having other diners and that they were interacting with each other. More detail could be included to bring more life to the space and sound effects would also help. |
| **4** | Details regarding the people weren't entirely true to life (that would probably fall into the uncanny valley if they were), but almost everything else felt pretty realistic. |
| **5** | The cafe was model well to reflect a real life cafe setting |
| **6** | pretty good representation of a small cafe. Liked the setup - adding some art could help. |
| **7** | It looked like a real restaurant and I felt like I was there - just the cartoony appearance of the patrons and staffs made me feel like I was inside a videogame |
| **8** | street should be a parking lot (like in a strip mall) not an ur |
| **Q5.** Was there any delay in rendering during head movement? | |
| **1** | no |
| **2** | NA |
| **3** | Some delay which could contribute to some feelings of nausea, especially with more extreme head movements. However, for this application (i.e. eating seated in a booth looking ahead) the head movement was minimal enough that it wasn't a big issue. |
| **4** | I didn't notice any delays. |
| **5** | I don't think there was a delay |
| **6** | only in situations where I broke through the set-up boundary ;-) |
| **7** | Everything was well-synched with my movements |
| **8** | no |
| **Q6.** How much did you feel a sense of being present in the virtual restaurant? | |
| **1** | feels like I am really there. |
| **2** | when looking around at the environment behind me, I felt more present |
| **3** | I felt more present when I moved around. |
| **4** | Because my job today was to evaluate the technology, I was removed a bit, but had the context been closer to what you'll have the participants do, I think I would have felt more present. |
| **5** | Felt fairly present. I think given the chance to look around and acclimate to the novelty will increase the sense of being present. |
| **6** | pretty present; |
| **7** | I felt like I really was inside a virtual and videogame-like restaurant. |
| **8** | it felt like a real restaurant. |
| **Q7.** Eating in a virtual restaurant is an appropriate methodology for measuring eating behavior when compared to traditional methods such as eating in a laboratory booth. | |
| **1** | maybe even moreso since a laboratory booth is dissimiliar to naturalistic eating environments. |
| **2** | It is appropriate depending on the research question |
| **3** | The appropriateness depends on the intended application. The benefits of feasibility and control offered by VR means it is a good method for exploration of eating behavior in different contexts. For studies with a standard context, a traditional lab booth is more appropriate. |
| **4** | I think it makes a lot of sense to investigate eating behavior through VR, assuming it provides the same perceived experience to every person as they would experience in a restaurant. It allows for much greater control while maintaining some ecological validity. |
| **5** | I think it could be |
| **6** | I feel this depends on the specific situation. It's more appropriate than a lab booth but less than in a real environment. |
| **7** | Although the ecological validity is not perfect, it definitely makes me think more of a restaurant than it would be if I were in a blank sensory booth |
| **8** | i am slightly skeptical of what questions can be asked but absolutely worth exploring. |
| **Q8.** Eating in a virtual restaurant provides contextual cues similar to those encountered when eating in a restaurant. | |
| **1** | just visual but not other sensory cues like hearing, smell. |
| **2** | i think the social contextual cues would be enhanced if they were more realistic (e.g. the person was interacting with the food in some way) |
| **3** | The VR restaurant included other people eating, workers surveying the space, and exposure to different food/drink cues such as the wall of wine bottles in the back. All of these are similar to what would be encountered in a real restaurant. |
| **4** | I agree that a VR eating environment like this provides more realistic contextual cues than eating in a booth might. |
| **5** | It is missing the pleasantness and comfort of a restaurant and the service component. |
| **6** | I think it could, but again, this would depend on the virtual environment as well as the specific person. I wonder about environment-by-individual interactions; some people might be just more or less prone to these virtual cues |
| **7** | Yes, I can imagine seeing advertisements for the special of the day and any other kind of cues I'd see in a real restaurant |
| **8** | agree that context is best use of VR |
| **Q9.** I was able to interact with food items without any restrictions. | |
| **1** | true except for needing to use straw |
| **2** | NA |
| **3** | (with the caveat that I didn't try drinking which might have offered problems) |
| **4** | The only problem I encountered was my spatial awareness while using the fork, but other than that, I didn't perceive any restrictions in my interactions. |
| **5** | No restriction in interacting, just the images changing with every hand movement is a little jarring. |
| **6** | yep, ate all the grapes |
| **7** | I could move and eat freely while inside the simulation |
| **8** | no issues eating. |
| **Q10.** The food in the pass through video looked as expected. | |
| **1** | color which is great, but color is much less vivid then real life. |
| **2** | the color/saturation of the food was a little different |
| **3** | It was clearly real food and looked like it, however the color was a bit dull and foods had a green glow around them. They were also a bit fuzzy/blurry and some things 'wobbled' with the lag from the headset. |
| **4** | The colors were ever so slightly mismatched and the video resolution appeared low, but I could tell that the food in front of me was real. |
| **5** | video looks very bright, so the food image seemed dull. |
| **6** | it looked like the real food; the food on the neighbor's plate however is another story. |
| **7** | The foods in the pass through maintained their shapes and their resolution was good enough to see them well, but their colors were not aligned with their shapes, especially as I moved my hands closer to my face. The shapes would appear black and white, and their colors would appear to the left of the shapes. |
| **8** | new headset was big improvement |
| **Q11.** How restrictive was the headset to your eating and drinking? | |
| **1** | only issue was needing to use straw. |
| **2** | using the straw was easy, as well as eating with my hand (grapes) and fork |
| **3** | (with the caveat that I didn't try drinking which might have offered problems) |
| **4** | Some foods, like the water cup with a straw or the finger food, were very easy to maneuver. When eating the pasta with sauce, I had a little more trouble, as I was concerned about bumping something with the headset while I leaned over to avoid spilling. |
| **5** | Not restrictive, but not super comfortable either. |
| **6** | I could drink and eat like normal |
| **7** | I could eat and drink without any problem or obstacles |
| **8** | no issue eating with fork hands or drinking with straw. drinking without a straw still an issue. |
| **Q12.** How restrictive was the hand interaction in restricting your eating and drinking? | |
| **1** | see above. |
| **2** | NA |
| **3** | There was basically a 1-1 match with where I was seeing my hand and what it was reaching for. |
| **4** | I didn't perceive any restriction. |
| **5** | I don't like the video moving in and out with hand movements |
| **6** | the one restricting issue was the non-overlapping circles. Bigger circles would help with this |
| **7** | The hand movement felt natural and didn't restrict my eating and drinking |
| **8** | na |
| **Q13.** How comparable was eating in the immersive restaurant compared to a "real world" restaurant | |
| **1** | feels natural but of course is not the same as the real thing since there is no sounds, smells, or human interaction. |
| **2** | unlike a restaurant, i did not have to place my order with a human or receive it from a human; it also lacked any social interaction that you might have with strangers at a restaurant (head nods, etc) |
| **3** | The VR environment had most of the elements of a real world environment, however everything was still clearly simulated and some of the visuals of the food in the real world window were 'off' in terms of color and clarity. |
| **4** | The "vibe" of eating in the VR restaurant was very similar to eating in a real restaurant, but there are some slight differences in realism. For instance, in a real restaurant, you would probably hear a conversation if the restaurant is small and relatively empty, but that isn't the case in the VR setting. |
| **5** | Comparable, but not the same |
| **6** | I mean it's for sure better and close to grabbing a meal by myself in a cafe. It's not comparable to eating in a proper sit-down restaurant with a waiter. |
| **7** | The VR did a good job at mimicking a real restaurant, despite the cartoony visual of the patrons and staff. The table being the same as the one in the lab adds a nice touch. The field of view disappearing and the displacement of color from shapes when moving the hands is the biggest factor that detracts from the realism of the experience. |
| **8** | na |
| **Q14.** If you were manipulating a virtual environment (such as a virtual restaurant) for a study, what would you add? What would you remove? | |
| **1** | add art. walls are barren. maybe some cars moving by outside. |
| **2** | background noise. sometimes restaurants have items sitting on the table that you can read (e.g. menus) that I might look at while waiting or eating. adding something like that could make it more realistic |
| **3** | I would add cutlery! I would also add some decor/ more detail to the space behind the table and add ambient chatter sound effects to bring some more life. You could also play with the food exposures in the space (e.g. the person next to you having different foods/portions, or the wall of wine replaced with a kitchen pass-through with different foods etc.) |
| **4** | I would probably add the soft din of voices, if possible. I don't think I would remove anything. |
| **5** | Music, food that matches the environment, art on the walls |
| **6** | as discussed, adding the neighbor briefly acknowledging me when I "enter" could help; adding some art on the wall behind me; doing something about the person showing her back to me to make them a bit more realistic could help (e.g., have them place their hands on the table, move there head a bit) |
| **7** | I'd add ambient sounds (in accordance with how busy the place seemed due to the number of patrons), and some decoration on the walls of the restaurant. I'd also make the test meal a bit closer to what you'd get in a restaurant. The combination of pasta with red sauce, grapes, and cookies feels more like lunch at a school cafeteria. |
| **8** | background noise! |
| **Q15.** If you were using this technology for a study, what food(s) and/or meal(s) would you serve? | |
| **1** | maybe have a realistic short menu with restaurant type food. |
| **2** | i think most foods would work except soup. penne is a little challenging because you have to line up the fork with a piece of pasta, I might try a food that is more amorphous or that is semi-solid but you can eat it with a spoon (orzo?) |
| **3** | Different entrees, different portions. Congruent and incongruent meals to the person eating next to me. |
| **4** | I would serve something that's easy to eat and not super messy. For example, I was worried that the pasta sauce would drip off of my fork onto the table or myself. You could potentially serve some kind of finger food, but I'm sure that would have a number of limitations, so probably just something without a sauce/dressing is what I would recommend, if possible. |
| **5** | Depends on the environment. The café looks like a place that would have sandwiches, soup, salad, and baked goods. I think the type of environment dictates the food served. The food served would need to be believable as a something that would be served in a restaurant. |
| **6** | depends on the study goal - I would probably stick with foods that can be eaten easily. one could also test which foods a person chooses and how that depends on whether they are having an easy or hard time eating in VR. |
| **7** | It would vary according with the environment being simulated for improved ecological validity. The types of food served in an a la carte restaurant are different from those served at a café and a cafeteria, for example. |
| **8** | unit foods like nuggets |
| **Q16.** If any, what are the advantages of eating in virtual reality compared to eating in a booth in the laboratory? | |
| **1** | can control and manipulate environment. possibly more naturalistic. |
| **2** | can simulate different situations, sounds, lighting |
| **3** | The ability to freely manipulate context and food cues is a huge benefit that practically is difficult to achieve in a lab booth. |
| **4** | It definitely provides a more realistic session for eating a meal, even if it's just simulated, while allowing researchers to maintain a great deal of control. |
| **5** | Pay less attention to manipulations that may be occurring because the environment is more stimulating. |
| **6** | the ability to change environmental cues easily is a plus; the ability to add and systematically change context |
| **7** | The ability to simulate different environments |
| **8** | same food in different contexts |
| **Q17.** If any, what are the disadvantages of eating in virtual reality compared to eating in a booth in the laboratory? | |
| **1** | novelty factor. possible dizziness. |
| **2** | unrealistic people/characteristics of the virtual environment could change eating in an unintended way; people could fixate on aspects of the virtual environment rather than eating their food as normal |
| **3** | It is not completely natural - there may be novelty effects or more distractions, the weight of the headset or any discomfort could influence eating behavior, the visuals of the foods through the window are not completely realistic in terms of sensory stimulation (specifically duller in color and blurry), and the lag in the headset might promote nausea. |
| **4** | The "uncanny valley"/lack of realism could deter some people from eating normally in a setting like this. It may also be hard for some to view VR eating as being the same experience as eating in real life. |
| **5** | The headset can be uncomfortable and the VR can cause little motion dizziness. |
| **6** | I think some people might not behave the same way, but then is the goal to replicate a lab booth or getting closer to a real life experience? |
| **7** | The way the pass through image interacts with your hands |
| **8** | headset is a little comfortable with glasses |
| **Q18.** To what extent do you think eating in virtual reality can replace current methods for researching eating behavior such as eating in a laboratory booth? | |
| **1** | I personally think the applications may be for very niche studies where youre mostly interested in how certain environemnt changes affect eating behavior. I dont see everyone adopting it without it serving a larger purpose for the design of the study. |
| **2** | i dont think it should replace other methods, but i think it can be used for research questions that require manipulating aspects of the environment |
| **3** | The benefits of feasibility of manipulating context offered by VR means it is a good method for exploration of eating behavior in different environments/with different cues. For more standard studies with more food focused manipulations I don't think it is a good replacement for a traditional lab booth. |
| **4** | If researchers want to learn more about how people eat or make food choices in the real world while desiring to maintain control over environmental factors, this seems like a good avenue that would be much more logistically practical than a real-world eating study. |
| **5** | I think they need to be tested and compared first, but opens a lot of options for adding in exposures or manipulating variables that are difficult to do in a normal lab setting. |
| **6** | I wonder how much of this is dependent on the individual - I think some might behave more naturally in VR than in a booth, others might do the opposite. The question then becomes what predicts how you behave in VR ... |
| **7** | I believe they might complement each other. Sensory booths are great for removing all possible variables and creating a very sterile experience, whereas the virtual reality will be great for adding more environments and food cues, and improving ecological validity |
| **8** | I think it will augment not replace |
| **Q19.** What would you change about the virtual reality experience to improve upon the experience? | |
| **1** | add art so less barren walls. I would appreciate sounds. |
| **2** | add background sound, make the person sitting next to me more realistic |
| **3** | Add more details to bring more life to the environment. Developments in tech will also help improve the limitations of the real-world window. |
| **4** | Not much, other than perhaps the sound of people's voices. The sound would have to match the approximate number of people in the restaurant though, which could prove challenging. |
| **5** | the video/images less jumpy or responsive to hand movements. Reduce any motion that may be bothersome |
| **6** | Depends on the goal, is the goal that everyone is fully immersed to a point where it's hard to differentiate VR from real? I feel this second iteration has improved so much, not sure if adding more context (e.g., sounds, smells) wouldn't lead to more distraction (unless that's the goal). |
| **7** | I'd add ambient sounds (in accordance with how busy the place seemed due to the number of patrons), and some decoration on the walls of the restaurant. I'd also make the test meal a bit closer to what you'd get in a restaurant. The combination of pasta with red sauce, grapes, and cookies feels more like lunch at a school cafeteria. |
| **8** | headset comfort, get rid of green halo on pass through |
| **Q20.** What benefits, if any, do you see in changing environments for eating behavior research? | |
| **1** | I think the environment plays a large role in eating behaviors so being able to control it in this manner opens up a lot of possibilities. |
| **2** | being about to assess the effects of different environments or cues |
| **3** | We know that different environments and cues can influence eating behavior. VR allows for more exploration of this. |
| **4** | It could help researchers translate findings from a lab/booth into a more realistic context. |
| **5** | Can reduce the impact of boredom or lack of engagement in laboratory studies. |
| **6** | I think it would allow tailoring environments to individual participants ... e.g., culture-specific contexts. |
| **7** | Understanding the effects of these environments |
| **8** | flexibility |
| **Q21.** Did the look of the food in the interface affect the taste of the food? | |
| **1** | no |
| **2** | because of the color difference between the interface and the real-world, there was increased uncertainty about what to expect for the first bite. But after that, the taste was not impacted |
| **3** | Not personally, but I could see it having an effect. |
| **4** | I did not perceive that, but I'm also a sensory scientist, so perhaps that bias was already absolved in my mind. |
| **5** | to some degree, the color of the VR foods is more vibrant than the real meal. |
| **6** | yes, I would not eat that vomit-looking mush of potatoes on the plate to the left of me. No, didn't feel it changed the taste of the food I ate. It's a bit odd that colors are pretty muted though - red grapes look dark, and tomato sauce on pasta looks not red but dark/brown |
| **7** | I don't think it affected the taste, but did make it a little peculiar to look at it due to the way the field of view changed based on my hand positioning and the distortions in shape/color overlapping |
| **8** | green halo wasnt great |
| **Q22.** Were there any difficulties with interacting with food items? | |
| **1** | no, besides the water situation. |
| **2** | no |
| **3** | No. |
| **4** | Aside from the fork, I didn't experience any difficulties. |
| **5** | Just the images moving with hand movements |
| **6** | nope |
| **7** | No |
| **8** | no |
| **Q23.** Did you experience motion sickness or any changes in nausea during your experience in VR? | |
| **1** | no. |
| **2** | no |
| **3** | A little bit when I was testing out the lag in the real word window by moving my head in different ways. When I was acting more naturally it was fine. |
| **4** | I did not experience any motion sickness. |
| **5** | Some |
| **6** | nope |
| **7** | None at all |
| **8** | no |
| **Q24.** Any additional comments. | |
| **1** | glad to see the technology improving. |
| **2** | no |
| **3** | Good job! |
| **4** | I'm excited to see where this research goes! |
| **5** | n/a |
| **6** | nope |
| **7** | This is impressive technology with very promising applications! |
| **8** | nope! |
